# CameraTransform: A Python package for perspective corrections and image mapping

**DOI:** 10.1016/j.softx.2019.100333

**Published:** 2019-10-03

**Authors:** Richard C. Gerum, Sebastian Richter, Alexander Winterl, Christoph Mark, Ben Fabry, Céline Le Bohec, Daniel P. Zitterbart

**Affiliations:** a Biophysics Group, Department of Physics, University of Erlangen-Nürnberg, Germany; b Applied Ocean Physics and Engineering, Woods Hole Oceanographic Institution, Woods Hole, MA, USA; c Centre Scientifique de Monaco, Département de Biologie Polaire, Principality of Monaco, Monaco; d Université de Strasbourg, CNRS, IPHC, UMR 7178, Strasbourg, France

**Keywords:** Perspective projection, Quantitative image analysis, Geo-referencing, Camera lens distortions

## Abstract

Camera images and video recordings are simple and non-invasive tools to investigate animals in their natural habitat. Quantitative evaluations, however, often require an exact reconstruction of object positions, sizes, and distances in the image. Here, we provide an open source software package to perform such calculations. Our approach allows the user to correct for perspective distortion, transform images to “bird’s-eye" view projections, or transform image-coordinates to real-world coordinates and vice versa. The extrinsic camera parameters that are necessary to perform such image corrections and transformations (elevation, tilt/roll angle, and heading of the camera) are obtained from the image using contextual information such as a visible horizon, GPS coordinates of landmarks, known object sizes, or images of the same object obtained from different viewing angles. All mathematical operations are implemented in the Python package *CameraTransform*. The performance of the implementation is evaluated using computer-generated synthetic images with known camera parameters. Moreover, we test our algorithm on images of emperor penguin colonies, and demonstrate that the camera tilt and roll angles can be estimated with an error of less than one degree, and the camera elevation with an error of less than 5%. The *CameraTransform* software package simplifies camera matrix-based image transformations and the extraction of quantitative image information. An extensive documentation and usage examples in an ecological context are provided at http://cameratransform.readthedocs.io.

**Table:** 

Code metadata	
Current code version	v1.1
Permanent link to code/repository used for this code version	https://github.com/ElsevierSoftwareX/SOFTX_2019_198
Legal Code License	MIT
Code versioning system used	git
Software code languages, tools, and services used	Python, Matplotlib, Pylustrator, SciPy, Numpy, Pandas
Compilation requirements, operating environments & dependencies	Python v3.4.x and higher
If available Link to developer documentation/manual	http://cameratransform.readthedocs.org/
Support email for questions	richard.gerum@fau.de

## 1. Introduction

Camera traps, time-lapse recordings, or video recordings are widely used tools in ecology research [1,2], for example for estimating abundance [3], or for behavioral studies [4,5]. However, such images inherently contain perspective distortions that need to be accounted for when accurate positions and distances need to be measured. To correct for perspective distortions and to map image points to real-world positions, it is paramount to know the intrinsic and extrinsic camera parameters [6,7]. Intrinsic parameters are the sensor and lens properties. Extrinsic parameters are the geographic camera position relative to landmarks in the scene, the camera elevation, tilt/roll angle, and heading. Some of the extrinsic parameters, however, are often difficult or impractical to measure in the field at the time of the recording. Here we present methods to reconstruct unknown extrinsic camera parameters from features in the image. The mathematical procedure behind this reconstruction is based on linear algebra and is implemented in the Python package *CameraTransform*. In addition to reconstructing extrinsic camera parameters, *CameraTransform* provides a toolbox to transform point coordinates in the image to geographic coordinates. In the following, we explain the mathematical details, estimate the reconstruction uncertainties, and describe practical applications.

## 2. Software

The complete software *CameraTransform*, released under the MIT license, is implemented in Python 3.6 [8], an interpreted programming language optimized for scientific purposes. For maximum efficiency, several open-source libraries are used. For numerical operations, such as matrix operations, we use the Numpy library [9]. Statistical distributions are implemented using the SciPy package [10]. The data are visualized using the Matplotlib [11] and Pylustrator [12] libraries, and are stored using the Pandas library [13].

## 3. Camera matrix

All information required for mapping real-world points (x, y, z coordinates in meters) to image points are stored in the camera matrix. The camera matrix is expressed in projective coordinates, and is split into two parts – the intrinsic matrix and the extrinsic matrix that correspond to the intrinsic and extrinsic camera parameters, respectively [14].

### 3.1. Intrinsic parameters

**Fig. 1.**
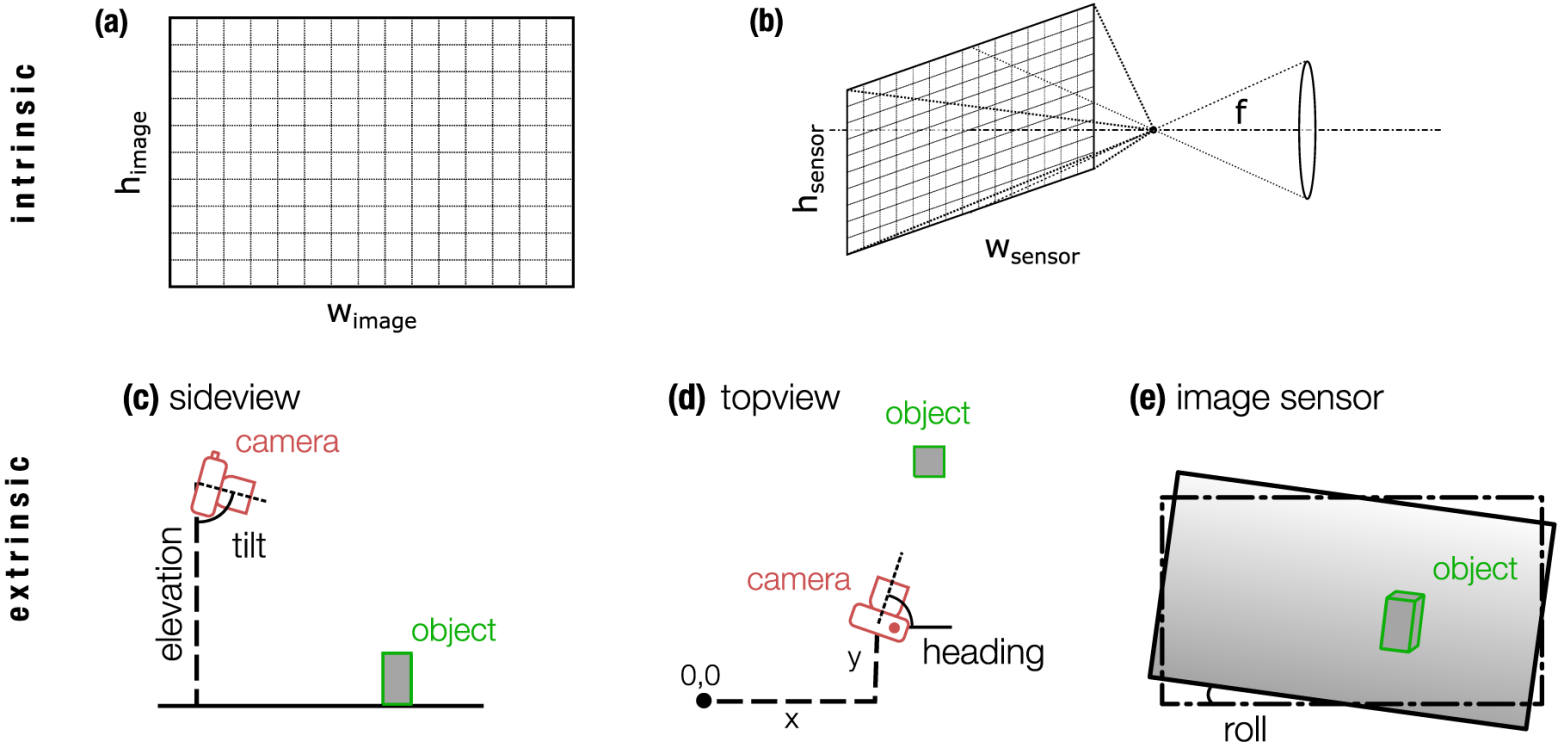
Camera parameters. Intrinsic parameters: (a) the dimensions of the image in pixels *w*_image_ × *h*_image_, (b) the size of the sensor in mm (*w*_sensor_ × *h*_sensor_) and the focal length *f* in mm. Extrinsic parameters: (c) side view: the *elevation* specifies the height of the camera above a reference altitude, e.g. above ground, the *tilt* specifies the angle between the vertical direction and the viewing direction (sensor normal). (d) top view: the *offset* (x, y) specifies the x, y coordinates of the camera relative to a reference position (*x* = 0, *y* = 0). and the *heading* specifies the angle between the *x*-direction and the viewing direction (sensor normal). (e) Image sensor: the *roll* specifies the angle between the lower sensor edge and the horizontal direction.

The intrinsic matrix entries contain information about the focal length f of the camera in mm, the effective sensor dimensions (*w*_sensor_ × *h*_sensor_) in mm, and the image dimensions (*w*_image_ × *h*_image_) in pixels (see Fig. 1a,b). Specifically, the intrinsic matrix entries are the effective focal length *f*_pix_ and the center of the sensor (*w*_image_/2, *h*_image_/2)

(1) $$C_{\text{intr}.}=\left(\begin{array}{cccc} f_{\text{pix}} & 0 & w_{\text{image}}∕2 & 0\\ 0 & f_{\text{pix}} & h_{\text{image}}∕2 & 0\\ 0 & 0 & 1 & 0 \end{array}\right)$$

(2) $$f_{\text{pix}}=f\cdot w_{\text{image}}∕w_{\text{sensor}}$$

The diagonal elements account for the re-scaling from pixels in the image to a position in mm on the sensor. The off-diagonal elements account for the offset between image and sensor coordinates, whereby the origin of the image is at the top left corner, and the origin of the sensor coordinates is at the center.

Eq. (1) only applies for the rectilinear projection of a pinhole camera (camera obscura). The *CameraTransform* package also supports cylindrical or equirectangular projections, which cannot be expressed in matrix notation. These are commonly used for panoramic images (see Supplementary Information). The package also supports corrections for radial lens distortions (see Supplementary Information).

### 3.2. Extrinsic parameters

The extrinsic matrix consists of the offset (x, y, z) of the camera relative to an arbitrary fixed real-world reference point (0,0,0). Customarily, (x = 0, y = 0) is the position of the camera, and the z-coordinate of the reference point is the ground level so that z is the elevation of the camera. The x, y plane of the coordinate system is usually the horizontal plane. Furthermore, the extrinsic matrix contains the tilt angle *α*_tilt_, the heading angle *α*_heading_, and the roll angle *α*_roll_ of the camera (see Fig. 1c-e and Supplementary Information).

The extrinsic parameters are used to rotate and translate the intrinsic camera matrix, with the aim to map or project 3-D points from real-world coordinates to 2-D image coordinates. The backprojection from a 2-D image point to 3-D real-world coordinates, however, is inherently underdetermined due to the lack of depth information in the image, and therefore requires one additional constraint, e.g. the z-coordinate of that real-world point or its distance to another real-world point. Supplementary Information explains several strategies to perform the rectilinear, cylindrical, or equirectangular backprojection.

## 4. Fitting of the extrinsic camera parameters

While the intrinsic camera parameters describing camera and lens properties are usually well known, this is often not the case for the extrinsic parameters that define the orientation of the camera. Below, we start with the simplest case where the heading and position of the camera is irrelevant and thus can be set to arbitrary values (e.g. 0). This leaves only three free parameters *elevation*, *tilt* and *roll*, unless the camera was properly horizontally aligned, in which case *roll* is approximately zero. The more complicated case where knowledge of camera heading and position is important, e.g. for multi-camera setups, or if the image needs to be geographically mapped, is described further below.

The *CameraTransform* package provides several fitting routines that allow the user to infer the extrinsic parameters from characteristic features in the image.

### 4.1. Fitting by object height

If the true height of objects in the image is known, for example for a group of animals, or more generally if distances between points seen in the image are known, the camera parameters can be fitted. This works especially well for the tilt angle as it most sensitively affects the apparent object height (see Supplementary Information, Fig. B.5b). To evaluate the fit parameter uncertainties, we use Metropolis Monte-Carlo sampling [16,17]. The input for this sampling process is a list of base (foot) and top (head) positions of the objects. Optionally, also the position of the horizon, landmarks, or reference objects such as rulers or survey poles can be provided to improve the algorithm (for details see below). The algorithm projects the foot positions from the image to real-world coordinates, using the constraint z = 0, then projects the head positions from the image to real-world coordinates, using as a constraint the x position of the projected foot points. The distance between these pairs of points is the estimated height of the object. This height is assigned a probability according to a known height distribution (e.g. the user does not need to know the exact height but can instead provide an estimate for the mean and standard deviation, or any non-Gaussian distributions of the expected object heights).

The Metropolis algorithm starts with arbitrarily chosen parameter values (for elevation, tilt, and roll) and evaluates the probability *p*_0_ assigned to these parameter values. Optionally, the user can provide starting values, but usually the algorithm converges well from a random initialization. Subsequently, small random numbers are added to the parameter values, and the corresponding probability *p*_1_ is re-evaluated. If the probability increases, the new parameter sample is saved, if the probability decreases, the new sample is only saved with a probability of *p*_1_/*p*_0_, otherwise discarded. After many such iterations (typically 10 000 in our case, which takes roughly 0.5 min on a standard desktop PC), the saved set of parameter samples represents the distribution of the fitted parameters (elevation, tilt, and roll). From these parameter samples, one can finally compute the mean value, denoting the best-fit parameter values, and the standard deviation or credible intervals, denoting the uncertainty of the parameters.

Optionally, if a horizon is visible in the image, *CameraTransform* uses the horizon line as an additional constraint to determine the extrinsic camera parameters. Based on the elevation, tilt and roll, the astronomical horizon line of a perfectly spherical earth is projected onto the image, and its distance to the user-provided horizon is minimized.

**Fig. 2.**
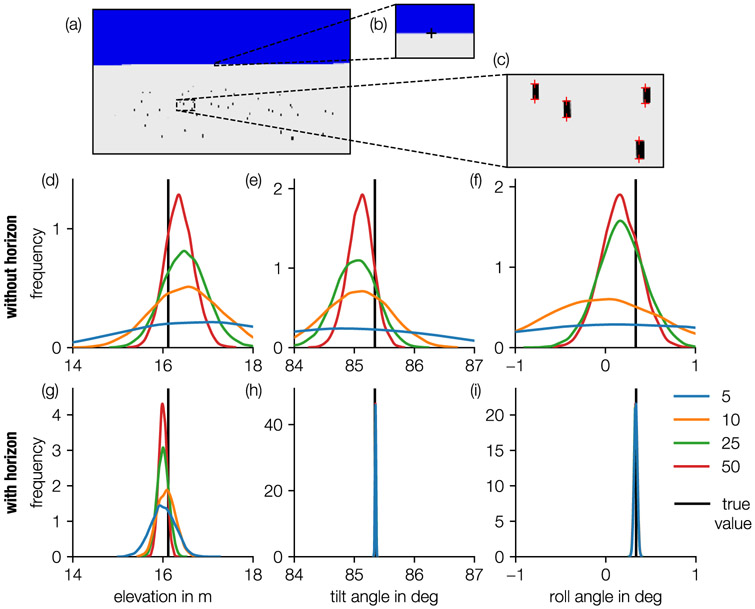
Influence of number of objects to determine camera elevation, tilt angle and roll angle. (a) Artificial image with randomly placed objects and horizon. (b) 3 points on the horizon and (c) foot and head points of 50 objects are manually selected. Metropolis-sampled uncertainty of the obtained parameters (elevation, tilt, and roll) for a fit without using horizon points (d)–(f) and with horizon points (g)–(i), for different number of randomly selected (without replacing) objects (N = 5, blue line; N = 10, orange line; N = 25, green line; N = 50 red line; true value, black line). (For interpretation of the references to color in this figure legend, the reader is referred to the web version of this article.)

To evaluate this method, an artificial image (Fig. 2a) is created using the *CameraTransform* package. A set of rectangles with a width of 30 cm and a height of 0.75m are randomly placed at distances ranging from 50m to 150 m, and subsequently projected to the image plane using the following camera parameters: focal length 14 mm, sensor size 17.3 × 9.7 mm with 4608 × 2592 px, camera height 16.1 m, tilt angle 85.3°, and roll angle 0.3°. Using the software *ClickPoints* [18], we mark the base and top positions of these rectangles in the image (Fig. 2c) and provide them as input for the sampling routine. We then investigate how the estimated distribution of elevation, tilt angle, and roll angle vary with the number of provided objects. The analysis is performed with and without a horizon, for 5, 10, 25, and 50 randomly chosen objects. The results show that by including a larger number *n* of objects, the uncertainty of the parameter estimate (as indicated by the width of the distribution) decreases roughly as *n*^−0.5^ (Fig. 2). Moreover, we find that both the camera elevation and the tilt angle can be fitted with considerably less uncertainty if a horizon is provided (Fig. 2g,h,i), compared to parameter estimates without horizon (Fig. 2d,e,f).

Furthermore, the uncertainty of the parameter estimates depends on the position of the objects in the image (see Supplementary Information). Objects that are evenly distributed throughout the image provide better estimates compared to objects that are clustered in the front or in the back of the image.

**Fig. 3.**
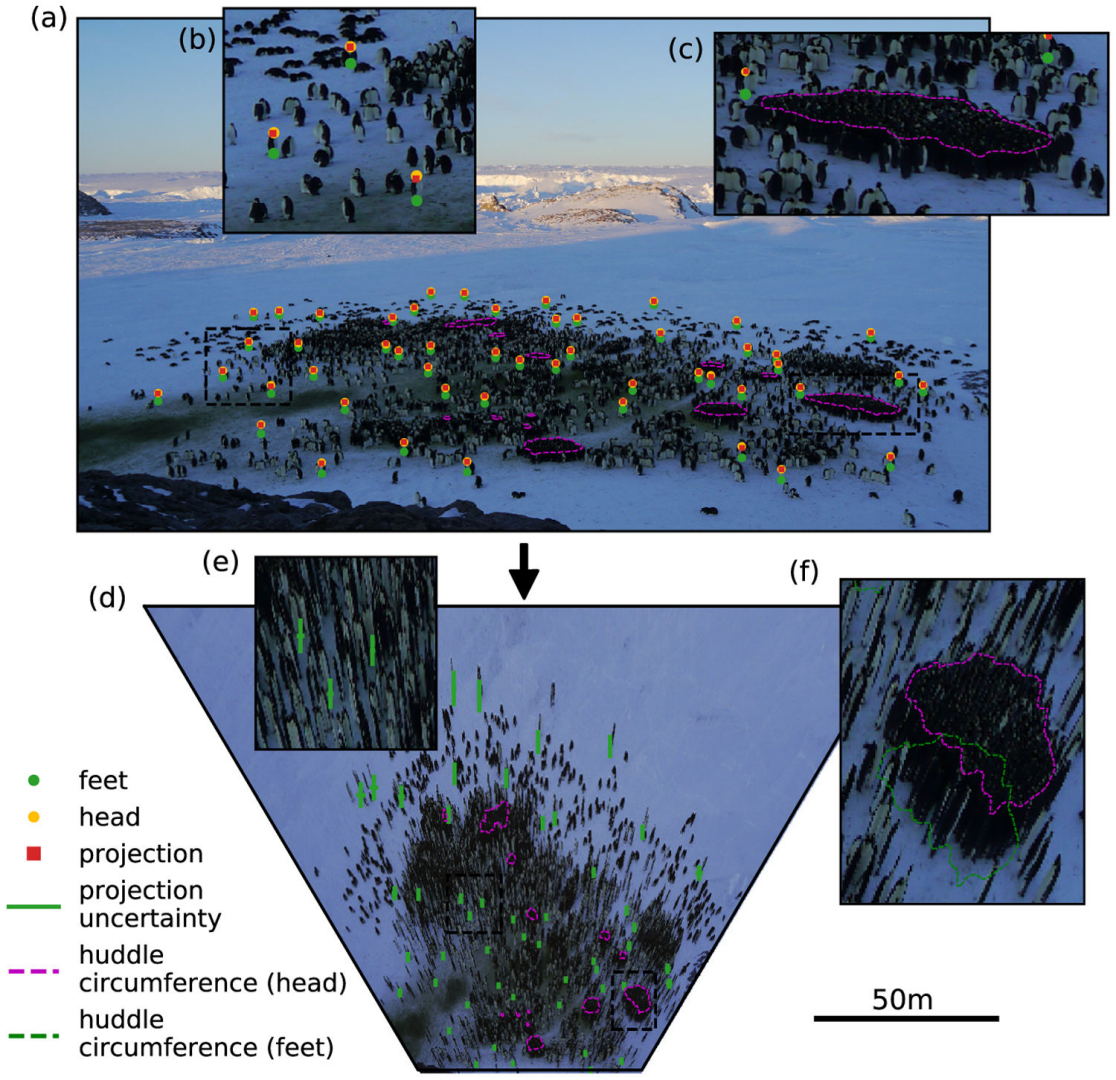
Fit of camera parameters using image objects. (a) Image taken with the micrObs system [15] of a penguin colony. The feet (green) and head (yellow) positions of 50 penguins were manually marked (shown in inset (b)) and the circumference of a huddle was marked (purple, shown in inset (c)). The head and feet positions are used to estimate the camera perspective (estimated head positions are shown as red squares) for projecting the image to a top view (d). Inset (e) shows the uncertainty of the projected penguin positions and inset (f) shows the projected huddle circumference.

To demonstrate the fitting procedure, we analyze an image (Fig. 3a) from a wide-angle camera overseeing an Emperor penguin (*Aptenodytes forsteri*) colony at Pointe Géologie, Antarctica (micrObs system, see [15]). The camera was positioned on an island with an elevation above sea ice level of 19m as measured by differential GPS. We estimate the extrinsic camera parameters by providing the feet and head positions of 50 animals, assuming an average height of 0.75m with an uncertainty that is left as a free parameter. Furthermore, we assume that the z-position of all animals is exactly equal as they are standing on the frozen sea ice, which we assume to be flat. Fig. 3b shows the projected top view based on the extracted extrinsic camera parameters. The estimated extrinsic parameters are: elevation 18.9 ± 0.7 m, tilt 84.6 ± 0.42°, and roll −0.2 ± 0.41°. The size variation of the penguins, which was left as a free parameter for the metropolis algorithm to sample, was estimated to be 0.059 m. The obtained parameters can now be used e.g. to estimate the area of huddles in the image. For this purpose, the user paints the region occupied by the heads of huddling penguins (pink line in Fig. 3a), and the circumference of this region is transformed to a top-view projection (pink line in Fig. 3d,f) and moved down by a distance of 0.75m (one penguin height) to indicate the huddle area (green line in Fig. 3d,f).

### 4.2. Fitting by geo-referencing

**Fig. 4.**
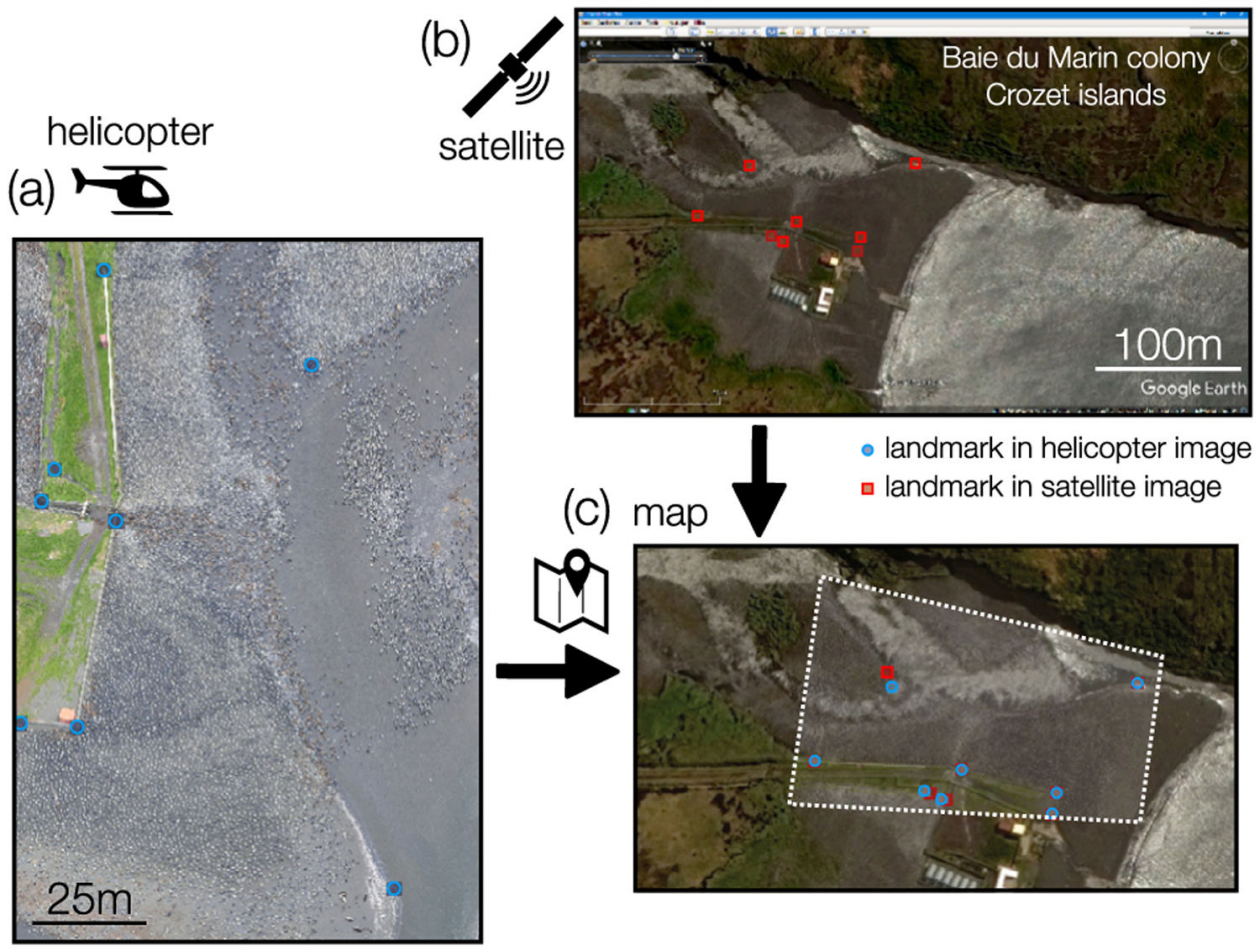
Fit of camera parameters by image registration. (a) Camera image from a helicopter flight at the Baie du Marin colony at the Crozet islands (Dec 09, 2014) [20]. (b) Satellite image provided by Google Earth. (c) Image fitted over points in the image (blue) with points in the map image (red). (For interpretation of the references to color in this figure legend, the reader is referred to the web version of this article.)

For small tilt angles, e.g. images taken from a helicopter (Fig. 4a), the size of the objects in the image does not vary sufficiently over the range of y-positions, and hence fitting by object height fails. If in addition there is no visible horizon, such images require a different method. If an accurate map or a high-resolution satellite image of the area of interest is available, point correspondences between the image and the map can be used instead to estimate the camera parameters using an image registration approach.

In the example shown in Fig. 4, photographs of a King penguin (*Aptenodytes patagonicus*) colony at the Baie du Marin (Possession Island, Crozet Archipelago) have been taken from a helicopter flying approximately 300m above ground. We choose eight distinct points that are recognizable in both the camera image and the satellite image provided by Google Earth (Fig. 4a,c). To calculate the cost function for image registration, we project the image points (blue points in Fig. 4a) onto the satellite image (blue points in Fig. 4c) and calculate the distance to the target points (red points in Fig. 4b,c). The fit routine of *CameraTransform* then computes the *height* and *tilt* of the camera as well as the camera’s x,y-position and heading. The example in Fig. 4 with an rms error of 0.76m between transformed image and target points demonstrates that the fit routine matches all except one point, which is the branch point of a river that has shifted from the time the satellite image was taken (Fig. 4c).

## 5. Stereo images

If object sizes are unknown, a stereo camera setup can be used to determine the size of multiple objects that are recorded from two different positions and angles, provided that corresponding points in both images can be unambiguously marked, and that either the distance between the two cameras or the absolute size of a single object in the image is known.

*CameraTransform* estimates the relative orientation of the cameras by minimizing the back-projected distance of a marked point in one image to the epipolar line of the corresponding point in the other image (the epipolar line is the line in image B that corresponds to a single point in image A), and vice versa (see Supplementary Fig. C7). We found that a minimization of the back-projection error in pixels is preferable to a more direct minimization of the distance between the corresponding rays of the points in the world space, due to scaling issues.

In contrast to existing stereo fitting methods (e.g. OpenCV), our method is based on the Monte Carlo approach and provides complete distributions of the estimated parameters to assess the uncertainty of the estimates. Furthermore, our method directly provides the relative orientation of the two cameras, whereas the commonly employed Eight-Point Algorithm [19] only yields the fundamental matrix, from which the relative orientation cannot be unambiguously extracted.

## 6. Impact

We present the Python package *CameraTransform* for estimating extrinsic camera parameters based on image features, satellite images, and images obtained from multiple viewing angles. Moreover, *CameraTransform* allows users to geo-reference images or to correct images for perspective distortions (e.g. to obtain top-view or “bird’s-eye” view projections). The package has been previously applied for studying Emperor and King penguin colonies [5,15,20], but is generally applicable for other quantitative image analysis where the extrinsic camera parameters were not or could not be measured in the field at the time of the recording. The package is published under the GPLv3 open source license to allow for continuous use and application in science. The documentation is hosted on http://cameratransform.readthedocs.io and contains further details on how to install the package. The documentation also provides numerous usage examples.

## Supplementary Material

1
