# Supplementary material for: CameraTransform: A Python package for perspective corrections and image mapping
Source: SoftwareX. Author manuscript; Available in PMC 2020 Aug 28. (PMC7453838; doi:10.1016/j.softx.2019.100333)
Supplement: 1 [file NIHMS1546289-supplement-1.pdf]

CameraTransform: a Python Package for Perspective Corrections and Image Mapping **Supplementary Material**

Richard C. Gerum[a], Sebastian Richter[a,b], Alexander Winterl[a,b], Christoph Mark[a], Ben Fabry[a], Céline Le Bohec[c,d], Daniel P. Zitterbart[a,b]

[a] *Biophysics Group, Department of Physics, University of Erlangen-Nürnberg, Germany*
[b] *Applied Ocean Physics and Engineering, Woods Hole Oceanographic Institution, Woods Hole, MA, USA*
[c] *Centre Scientifique de Monaco, Département de Biologie Polaire, Monaco, Principality of Monaco*
[d] *Université de Strasbourg, CNRS, IPHC, UMR 7178, Strasbourg, France*

Keywords: Perspective projection, quantitative image analysis, geo-referencing, camera lens distortions

Appendix A. Camera Matrix

All information about the mapping of real-world points to image points are stored in a camera matrix. The camera matrix is expressed in projective coordinates, and can be split into two parts - the intrinsic matrix and the extrinsic matrix [1]. The intrinsic matrix depends on the camera sensor and lens, the extrinsic matrix depends on the camera's position and orientation.

Appendix A.1. Projective coordinates

Projective coordinates, also known as homogeneous coordinates, are used to represent projective transformations as matrix multiplications [2] whereby the vector representation of a point is extended by an additional entry. This entry defaults to 1, and all scalar multiples of a vector are considered equal:

$$\begin{pmatrix} x \\ y \\ 1 \end{pmatrix} \hat{=} \begin{pmatrix} s \cdot x \\ s \cdot y \\ s \end{pmatrix} \tag{A.1}$$

For example, the point (5,7) can be represented by the tuple of projective coordinates (5,7,1) or (10,14,2) and so on. The scalar s need not be an

integer. Projective coordinates allow us to write the camera projection $\vec{y}$ as:

$$\begin{pmatrix} y_1 \\ y_2 \\ 1 \end{pmatrix} = \begin{pmatrix} c_{11} & c_{12} & c_{13} & c_{14} \\ c_{21} & c_{22} & c_{23} & c_{24} \\ c_{31} & c_{32} & c_{33} & c_{34} \end{pmatrix} \cdot \begin{pmatrix} x_1 \\ x_2 \\ x_3 \\ 1 \end{pmatrix} \tag{A.2}$$

where $\vec{x}$ specifies the point in the 3D world, which is multiplied with the camera matrix C to obtain the point in the camera image $\vec{y}$.

Appendix A.2. Intrinsic parameters

The intrinsic parameters are given by Equation (1) & (2) in the main text.

Appendix A.3. Extrinsic parameters

To compute the extrinsic camera matrix, we first need the three rotation matrices and the translation matrix:

$$R_{\text{tilt}} = \begin{pmatrix} 1 & 0 & 0 \\ 0 & \cos(\alpha_{\text{tilt}}) & \sin(\alpha_{\text{tilt}}) \\ 0 & -\sin(\alpha_{\text{tilt}}) & \cos(\alpha_{\text{tilt}}) \end{pmatrix} \tag{A.3}$$

$$R_{\text{roll}} = \begin{pmatrix} \cos(\alpha_{\text{roll}}) & \sin(\alpha_{\text{roll}}) & 0 \\ -\sin(\alpha_{\text{roll}}) & \cos(\alpha_{\text{roll}}) & 0 \\ 0 & 0 & 1 \end{pmatrix} \tag{A.4}$$

$$R_{\text{heading}} = \begin{pmatrix} \cos(\alpha_{\text{heading}}) & \sin(\alpha_{\text{heading}}) & 0 \\ -\sin(\alpha_{\text{heading}}) & \cos(\alpha_{\text{heading}}) & 0 \\ 0 & 0 & 1 \end{pmatrix} \tag{A.5}$$

$$t = \begin{pmatrix} x \\ y \\ -\text{height} \end{pmatrix} \tag{A.6}$$

$$\tag{A.7}$$

The extrinsic camera matrix then consists of the 3x3 rotation matrix R and the 3x1 translation matrix t side by side, as a 4x4 matrix in projective coordinates.

$$R = R_{\text{roll}} \cdot R_{\text{tilt}} \cdot R_{\text{heading}} \tag{A.8}$$

$$T = R_{\text{tilt}} \cdot R_{\text{heading}} \cdot t \tag{A.9}$$

$$C_{\text{extr.}} = \left(\begin{array}{c|c} R & T \\ \hline 0 & 1 \end{array}\right) \tag{A.10}$$

The final camera matrix C is the product of the intrinsic and the extrinsic camera matrix.

$$C = C_{\text{intr.}} \cdot C_{\text{extr.}} \tag{A.11}$$

Appendix A.4. Projecting from the World to the Camera image

To map a real-world point to a pixel of the acquired image, we first write the real-world point $\vec{p}_{\text{world}}(x_1, x_2, x_3)$ in projective coordinates:

$$\tilde{p}_{\text{world}} = \begin{pmatrix} x_1 \\ x_2 \\ x_3 \\ 1 \end{pmatrix} \tag{A.12}$$

The image point $\tilde{p}_{\text{im}}$ can then be computed according to:

$$\tilde{p}_{\text{im}} = C \cdot \tilde{p}_{\text{world}} \tag{A.13}$$

Finally, the point $\tilde{p}_{\text{im}}$ is converted from projective coordinates (which has 3 entries) to "conventional" coordinates $\vec{p}_{\text{im}}$ (with two entries) by division with the additional scaling factor s (which is the 3rd entry of $\tilde{p}_{\text{im}}$):

$$\vec{p}_{\text{im}} = \begin{pmatrix} \tilde{p}_{\text{im}_1} / \tilde{p}_{\text{im}_3} \\ \tilde{p}_{\text{im}_2} / \tilde{p}_{\text{im}_3} \end{pmatrix} \tag{A.14}$$

where the subscript denotes the entry of the vector $\tilde{p}_{\text{im}}$.

Appendix A.5. Projecting from the camera image to real-world coordinates

While projecting from the 3D real-world to the 2D image is a straight forward matrix multiplication, projecting from the image back to the real-world is more difficult. As the information of the 3rd dimension is lost during the transformation from the real-world to the image, there exists no unique back-transformation. An additional constraint is needed to transform a point back to the 3D world, e.g. one of the 3D coordinates must be fixed. For example, if the real-world point $\vec{p}_{\text{world}}$ has a known x_3 coordinate (e.g. the height above ground is known), and the image coordinates y_1 and y_2 are given, the back-transformation can be performed as follows:

$$\begin{pmatrix} y_1 \\ y_2 \\ 1 \end{pmatrix} = \begin{pmatrix} c_{11} & c_{12} & c_{13} & c_{14} \\ c_{21} & c_{22} & c_{23} & c_{24} \\ c_{31} & c_{32} & c_{33} & c_{34} \end{pmatrix} \cdot \begin{pmatrix} s \cdot x_1 \\ s \cdot x_2 \\ s \cdot x_3 \\ s \end{pmatrix} \tag{A.15}$$

$$= \begin{pmatrix} c_{11} & c_{12} & c_{13} \cdot x_3 & c_{14} \\ c_{21} & c_{22} & c_{23} \cdot x_3 & c_{24} \\ c_{31} & c_{32} & c_{33} \cdot x_3 & c_{34} \end{pmatrix} \cdot \begin{pmatrix} s \cdot x_1 \\ s \cdot x_2 \\ s \\ s \end{pmatrix} \tag{A.16}$$

$$= \begin{pmatrix} c_{11} & c_{12} & c_{13} \cdot x_3 + c_{14} \\ c_{21} & c_{22} & c_{23} \cdot x_3 + c_{24} \\ c_{31} & c_{32} & c_{33} \cdot x_3 + c_{34} \end{pmatrix} \cdot \begin{pmatrix} s \cdot x_1 \\ s \cdot x_2 \\ s \end{pmatrix} \tag{A.17}$$

$$= \tilde{C} \begin{pmatrix} s \cdot x_1 \\ s \cdot x_2 \\ s \end{pmatrix} \tag{A.18}$$

$$\tilde{C}^{-1} \cdot \begin{pmatrix} y_1 \\ y_2 \\ 1 \end{pmatrix} = \begin{pmatrix} s \cdot x_1 \\ s \cdot x_2 \\ s \end{pmatrix} \tag{A.19}$$

This means that the information about the fixed 3D coordinate has to be incorporated in the camera matrix. The inverse of the resulting matrix, when multiplied with the image point in projective coordinates, gives the unknown x_1 and x_2 entries of the real-world 3D point. After re-scaling the vector entries (division by s), the known x_3 value is added to the vector to retrieve the real-world coordinates of the 3D point $\vec{p}_{\text{world}}$.

For transformations that cannot be expressed as a matrix, the ray of the given pixel is obtained, then transformed with the extrinsic matrix, and finally the intersection of this ray with e.g. a coordinate plane is calculated.

Appendix A.5.1. Rectilinear Projection

This rectilinear projection is the standard "pin-hole" camera model, which is the most common projection for single images. The 3D point (x, y, z) is projected on a plane $(x_{\text{im}}, y_{\text{im}})$. The transformations here are given for a camera pointing in z direction. For an arbitrary orientation of the camera, the 3D point has to be first transformed to account for the camera's position and orientation.

$$x_{\text{im}} = f_x \cdot \frac{x}{z} + x_{\text{offset}} \tag{A.20}$$

$$y_{\text{im}} = f_y \cdot \frac{y}{z} + y_{\text{offset}} \tag{A.21}$$

Where f_x and f_y are the focal lengths in pixel for the x and y direction (for an ideal camera with square pixels, f_x and f_y are equal). The offsets account for the origin (0,0) of the image usually being the top left corner and not the center of the image.

Because information is lost when projecting from 3D to 2D, there is no unique backtransformation. For every 2D point, however, a unique line in 3D space can be specified on which the 3D point lies. This so called "ray" is defined as follows:

$$\vec{r} = \begin{pmatrix} (x_{\text{im}} - x_{\text{offset}})/f_x \\ (y_{\text{im}} - y_{\text{offset}})/f_y \\ 1 \end{pmatrix} \tag{A.22}$$

Appendix A.5.2. Cylindrical Projection

The cylindrical projection is a common projection used for panoranic images. The 3D point is projected on the 2D surface of a cylinder. This projection is often used for wide panoramic images, as it can cover the full 360°range in the x-direction. The poles, however, cannot be represented in this projection.

$$x_{\text{im}} = f_x \cdot \arctan\left(\frac{x}{z}\right) + x_{\text{offset}} \tag{A.23}$$

$$y_{\text{im}} = f_y \cdot \frac{y}{\sqrt{x^2 + z^2}} + y_{\text{offset}} \tag{A.24}$$

The image rays are defined as follows:

$$\vec{r} = \begin{pmatrix} \sin\left(\frac{x_{\text{im}} - x_{\text{offset}}}{f_x}\right) \\ \frac{y_{\text{im}} - y_{\text{offset}}}{f_y} \\ \cos\left(\frac{x_{\text{im}} - x_{\text{offset}}}{f_x}\right) \end{pmatrix} \tag{A.25}$$

Appendix A.5.3. Equirectangular Projection

The equirectangular projection is a common projection used for panoranic images. The 3D point is projected on the 2D surface of a sphere. The projection can cover the full range of angles in both x and y direction.

$$x_{\text{im}} = f_x \cdot \arctan\left(\frac{x}{z}\right) + x_{\text{offset}} \tag{A.26}$$

$$y_{\text{im}} = f_y \cdot \arctan\left(\frac{y}{\sqrt{x^2 + z^2}}\right) + y_{\text{offset}} \tag{A.27}$$

The image rays are defined as follows:

$$\vec{r} = \begin{pmatrix} \sin\left(\frac{x_{\mathrm{im}} - x_{\mathrm{offset}}}{f_x}\right) \\ \tan\left(\frac{y_{\mathrm{im}} - y_{\mathrm{offset}}}{f_y}\right) \\ \cos\left(\frac{x_{\mathrm{im}} - x_{\mathrm{offset}}}{f_x}\right) \end{pmatrix} \tag{A.28}$$

Appendix B. Sensitivity Analysis

Appendix B.1. Extrinsic Camera parameters

To evaluate the sensitivity of the perspective projection with respect to uncertainties in the camera parameters, we computationally place objects of 1 m height in world coordinates at different distances from the camera (50 – 300 m) and project them to the camera image. The positions in the camera image are then projected back to real-world coordinates using a different parameter set where we vary the camera elevation and tilt angle. We use a *Panasonic DMC-G5* camera with a focal length of 14 mm and a sensor size of 17.3×9.7 mm with 4608×2592 px. The camera is placed at an elevation of 20 m with a tilt angle of 80°. For the back projection, the elevation and tilt are varied by ±10% (Fig. B.1) and for each parameter configuration the apparent object height is calculated. Since we know the true object height, the reconstructed object height indicates the error that is introduced by the uncertainties in the extrinsic camera parameters. We find that the apparent object height is only weakly dependent on variations in camera elevation regardless of the distance between object and camera (Fig. B.1b). By contrast, the apparent object height is sensitive to variations in the camera's tilt angle, especially for objects with a larger distance to the camera (Fig. B.1c).

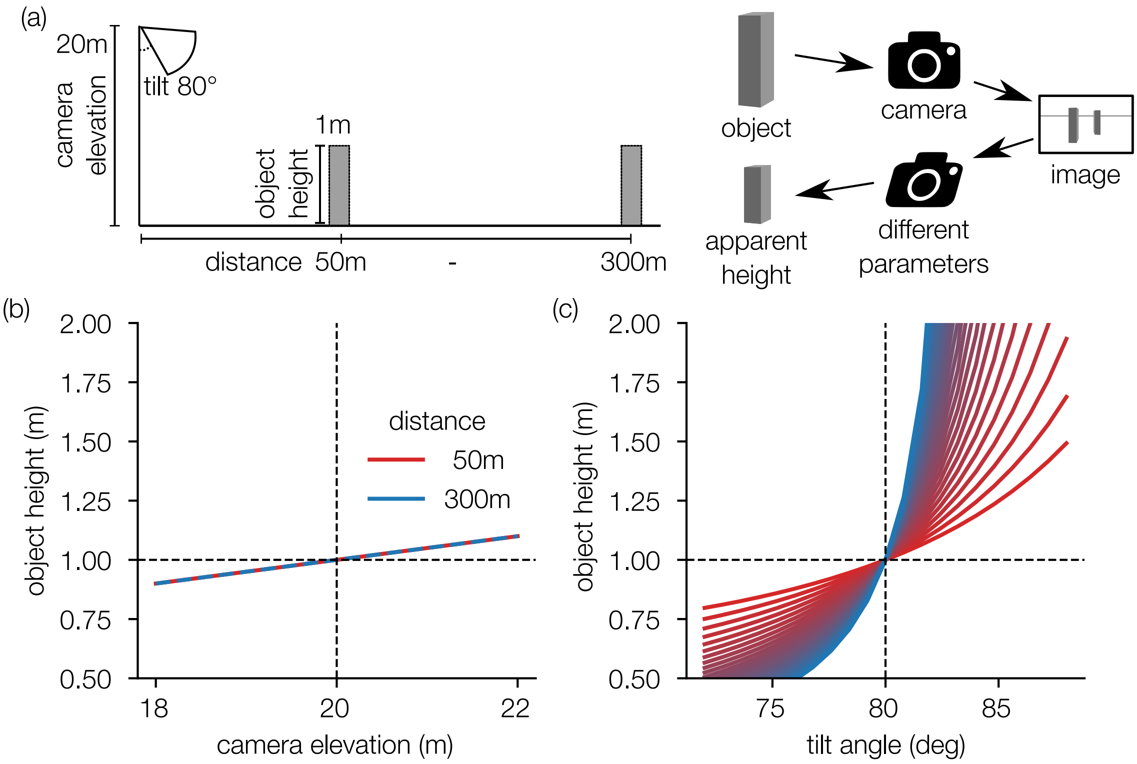

Figure B.1: **Influence of elevation and tilt angle variation of ± 10%.** (a) Objects with a height of 1 m (dashed line, in (b),(c)) and different distances (50 m – 300 m) projected to the camera and back to the world with changed camera parameters. (b) Object height for variation of the elevation parameter (20 m ± 10%). (c) Object height for variation of the the tilt parameter (80°± 10%).

Appendix B.2. Object positions

Fitting the camera parameters from objects in the image not only depends on the number of objects used to estimate the camera parameters, but also on the position of the objects in the image. As an estimate of this dependence, we use an artificially create image with known camera parameters (focal length 3863.64 px, image 2592 x 4608 px, elevation 16.12 m, tilt 85.3°, roll 0.34°), where we place 50 objects at different distances from the camera (between 46 m and 151 m).

Using the objects in the foreground yielded better results (smaller uncertainty) for the elevation parameter than using the objects in the background of the image (see Fig. B.2). For the tilt and roll parameters, this behaviour is reversed. Using objects from both foreground and background for the fitting routine, by contrast, gave the best results for all parameters.

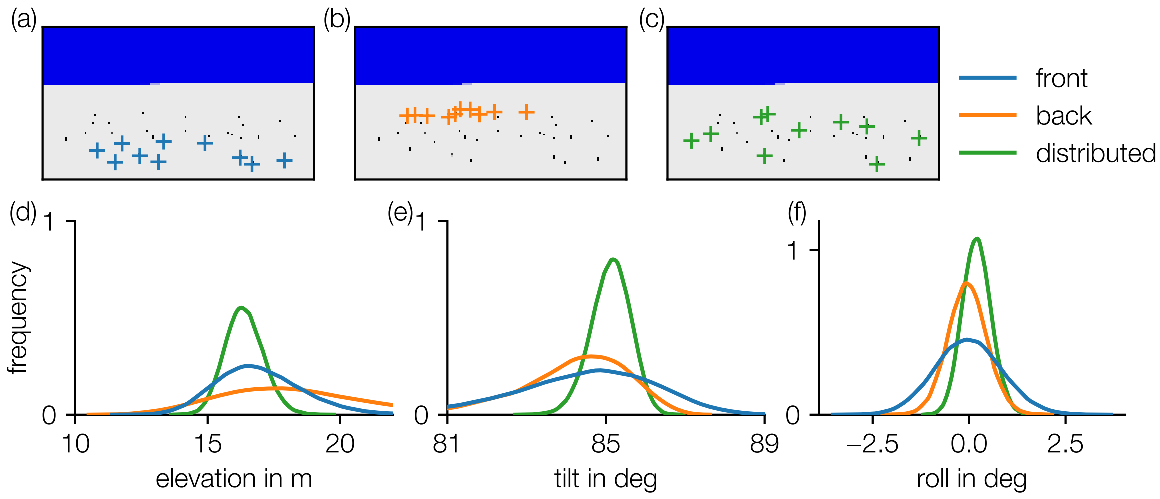

Figure B.2: **Influence of the position of the provided objects on the parameter uncertainty.**
On an artificial image (known camera parameters) with 50 objects where the foot and head positions are manually marked. For every analysis, 10 objects are used. The 10 nearest objects (blue, shown in (a)), the 10 farthest objects (orange, shown in (b)) and 10 randomly distributed objects (green, shown in (c)). (d)-(f) The uncertainty of the camera parameters (elevation, tilt, and roll) for the different conditions.

Appendix C. Fitting from Stereo Image

To test the reconstruction of the camera parameters from stereo images, two sample images (Fig. C.3g,h) of a table with various objects of known size are taken with a camera that is laterally moved between the images. A total of 16 point correspondences in the two images are then marked, which serve as the input for the metropolis sampling algorithm. During sampling, the baseline (distance between the two cameras) remains fixed to unity and is later re-scaled to fit the known sizes of the objects in the images, resulting in a fitted baseline of 87 cm. This re-scaling is essential as point correspondences alone cannot provide information on scaling. For the sampling, a total of 5 parameters (camera A: heading, roll; camera B: tilt, heading, roll) are sampled, while the other parameters (camera A: position (x=0, y=0, z=0), heading = 90°; camera B: position (x=1, y=0, z=0)) are fixed to define a unique reference frame.

After sampling, distances and object sizes in the image are measured by assigning corresponding points in the stereo images. The calculated sizes and distances are then verified with a ruler and are found to be accurate to within ± 1 mm.

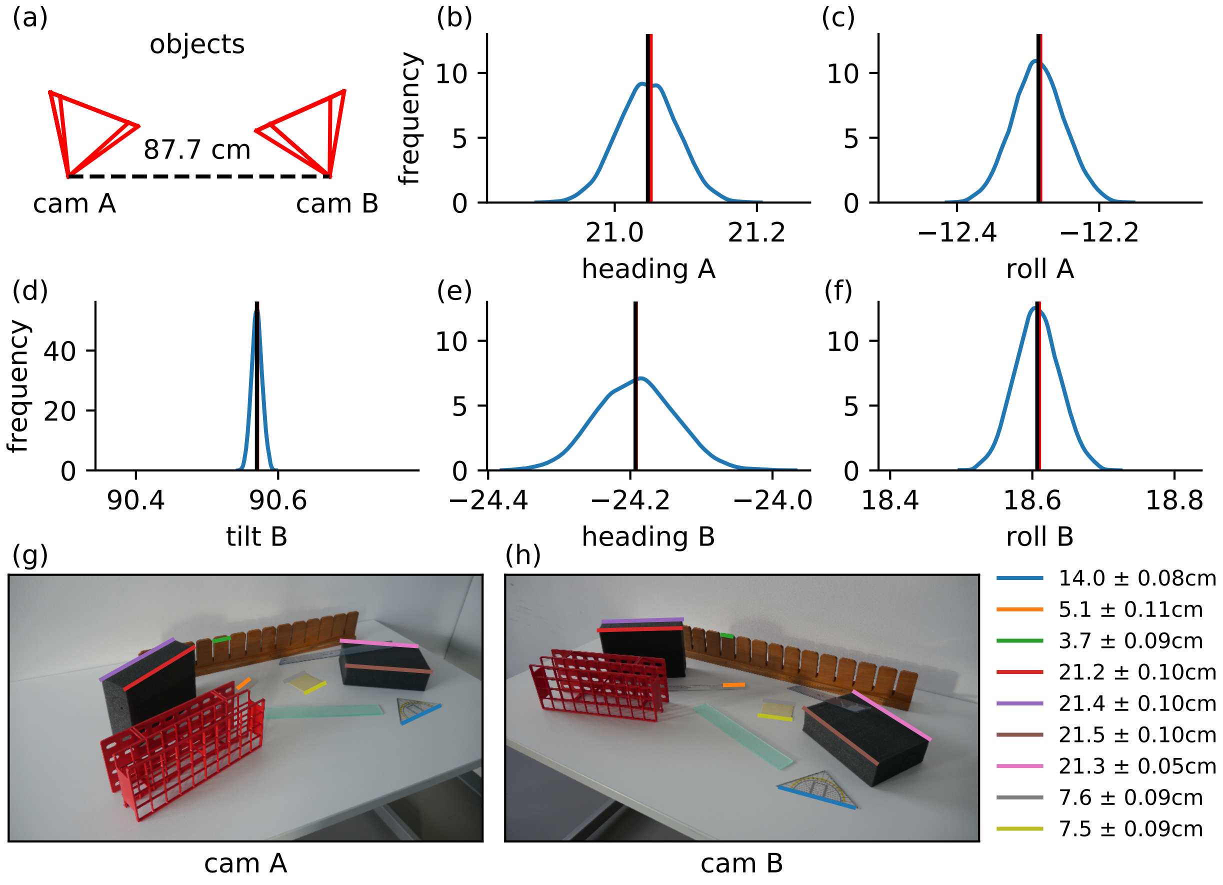

Figure C.3: **Fit of camera parameters by point-correspondences in a stereo setup.**
(a) Setup of the two cameras. (b,c,d,e,f) The fitted distributions of the parameter uncertainties for camera A (b,c) and camera B (d,e,f). (g) Image of the left camera and image of the right camera (h). Colored lines show distances that have been measured using point correspondences.

Appendix D. Lens distortions

The accuracy of the image transformation package can be further improved by considering image distortions caused by imperfect camera lenses. *CameraTransform* is currently able to deal with radial lens distortions but not skew and tangential distortions that are usually less severe. *CameraTransform* directly removes lens distortions when projecting from image coordinates to world coordinates without the need to first compute an undistorted image, which would introduce rounding and pixelation errors. Conversely, it is also possible to apply lens distortions when back-projecting from world coordinates to image coordinates.

CameraTransform implements two lens distortion models, the commonly used Brown model and the ABC model. The Brown model moves image points along the line formed by the image point and the optical axis point radially according to a polynomial function of the radial distance with even polynomial powers. This distortion model is also used e.g. by OpenCV or AgiSoft PhotoScan. The ABC model uses a 4th order polynomial for the radial shift and is used e.g. in PTGui.

References

[1] R. Hartley, A. Zisserman, Multiple view geometry in computer vision, Cambridge University Press, Cambridge, 2003 (2003).

[2] A. F. Möbius, Der barycentrische Calcul, ein Hülfsmittel zur analytischen Behandlung der Geometrie, Barth, Leipzig, 1827 (1827).
